# The time course of auditory recognition measured with rapid sequences of short natural sounds

**DOI:** 10.1038/s41598-019-43126-5

**Published:** 2019-05-29

**Authors:** Vincent Isnard, Véronique Chastres, Isabelle Viaud-Delmon, Clara Suied

**Affiliations:** 1 Département Neurosciences et Sciences Cognitives, Institut de Recherche Biomédicale des Armées, Brétigny sur Orge, France; 2 Equipe Espaces Acoustiques et Cognitifs, Institut de Recherche et Coordination Acoustique/Musique, Sciences et Technologies de la Musique et du Son UMR, 9912 Paris, France; 3 Laboratoire de Biophysique Neurosensorielle, UFR de Médecine, Université Clermont Auvergne, Neuro-Dol UMR INSERM 1107, Clermont-Ferrand, France

**Keywords:** Cortex, Human behaviour

## Abstract

Human listeners are able to recognize accurately an impressive range of complex sounds, such as musical instruments or voices. The underlying mechanisms are still poorly understood. Here, we aimed to characterize the processing time needed to recognize a natural sound. To do so, by analogy with the “rapid visual sequential presentation paradigm”, we embedded short target sounds within rapid sequences of distractor sounds. The core hypothesis is that any correct report of the target implies that sufficient processing for recognition had been completed before the time of occurrence of the subsequent distractor sound. We conducted four behavioral experiments using short natural sounds (voices and instruments) as targets or distractors. We report the effects on performance, as measured by the fastest presentation rate for recognition, of sound duration, number of sounds in a sequence, the relative pitch between target and distractors and target position in the sequence. Results showed a very rapid auditory recognition of natural sounds in all cases. Targets could be recognized at rates up to 30 sounds per second. In addition, the best performance was observed for voices in sequences of instruments. These results give new insights about the remarkable efficiency of timbre processing in humans, using an original behavioral paradigm to provide strong constraints on future neural models of sound recognition.

## Introduction

Anecdotally, we as human listeners seem remarkably apt at recognizing sound sources: the sound of a voice, approaching footsteps, or musical instruments in each of our cultures. There is now quantitative behavioral evidence supporting this idea (for a review, see Agus *et al*., in press^1^). However, the underlying neural mechanisms for such an impressive feat remain unclear.

One way to constrain the range of possible mechanisms is to measure the temporal characteristics of sound source recognition. Using a straight-forward operational definition of recognition as a correct response to a target sound defined by its category (e.g., a voice among musical instruments), Agus *et al*.^2^ have shown that reaction times for recognition were remarkably short, with an overhead compared to simple detection between 145 ms and 250 ms depending on target type. When natural sounds were artificially shortened by applying an amplitude “gate” of variable duration, it was observed that recognition remained above chance for durations in the milliseconds range^3–5^. However, none of these results speak directly to the processing time required for sound recognition. For reaction times, the comparison of recognition and simple detection times cannot be unequivocally used to estimate processing time^6^. For gating, recognizing a very short sound presented in isolation could still require a very long processing time: the short sound duration only constrain the type of acoustic features that are used^7^.

Similar questions about the processing time required for visual recognition of natural objects have been asked^8,9^. They have typically been addressed by what is known as the now-classic “rapid sequential visual presentation task” (RSVP)^10–13^ (for a review, see Keysers *et al*.^13^). Briefly, in RSVP, images are flashed in a rapid sequence, with images from one target category presented among many distractors belonging to other categories. Participants are asked to report the sequences containing an image from the target category. The fastest presentation rate for which target recognition remains accurate is taken as a measure of processing time. The core hypothesis is that, for a target to be accurately recognized, it needs to have been sufficiently processed before the next distractor is flashed^13^.

Using a new auditory paradigm inspired by RSVP, the Rapid Audio Sequential Presentation paradigm (RASP; see Suied *et al*.^14^ for pilot data), the current study addresses the processing time of natural sounds like voices and instruments. Natural sounds were presented in rapid succession and participants had to report sequences containing a sound from a target category (e.g., a voice) among several distractor sounds from other categories (e.g., musical instruments). Unlike for vision, in audition sounds cannot be flashed instantaneously. Fortunately, gating studies have shown that short sounds were still recognizable^5^, so we used short gated sounds to create sufficiently rapid sequences. The experimental measure used is the fastest presentation rate for which the task could be performed above chance. This measure was taken as an estimate for the processing time needed for recognition.

Temporal processing is a vast field of inquiry for audition, with time constants found in the range of microseconds for sound localization^15^ up to seconds for acoustic memory^16^ and includes variable windows in the tens of milliseconds for e.g. pitch^17,18^ or speech perception^19^. However, there are very little previous studies that aimed to characterize processing times with methods and intent similar to RASP. In seminal studies using backward masking, which can be viewed as a RASP paradigm for a sequence of single target – single distractor, the duration of a so-called “echoic memory” was evaluated (reviewed by e.g. Massaro^20^; Cowan^21^). When a brief target was followed by a second masking sound, recognition of the target sound reaches a plateau with an inter-onset interval of about 250 ms. This duration was hypothesized to correspond to the duration of a memory store used for auditory recognition^20^. In another series of experiments on sequence processing, Warren and colleagues measured the fastest sequences for which processing was local, i.e. for each individual sound, instead of global at the sequence level, as indexed for instance by order discrimination tasks^22–24^. Overall, they advocated for a global mode of processing when sequence items were shorter than about 40 ms.

Finally, in some of our previous work, the RASP paradigm was introduced^14^. Sequences of short distractor sounds were presented with, in half of the trials, a short target sound at a random position in the sequence. Performances decreased with increased presentation rate, but recognition was still possible for presentation rates of up to 30 sounds per second. This suggests a lower-bound limit for processing time needed for recognition as short as 30 ms. This seems remarkable because, even with the high temporal resolution of electrophysiological recordings, a minimum of 70 ms after stimulus onset had been needed to observe differences in evoked related potentials (ERPs) between categories of sounds^25,26^. However, this first use of a RASP paradigm was more a proof of concept than an extensive mapping of recognition processing time. In the present study, the RASP paradigm was used to further investigate the lower-bound limit of natural sound recognition put forward by Suied *et al*.^14^ and test for a variety of potential interpretations.

We used a sound corpus presenting on the one hand a large acoustical variability, but on the other hand attempted to globally match as much as possible the target and distractor categories. As in Agus *et al*.^2^ and Suied *et al*.^5^, we used sung vowels and musical instruments as stimuli. All were musical sounds so they could be drawn from the same pitch range, making pitch an irrelevant cue for recognition and forcing participants to use timbre cues (all stimuli were presented with the same duration and loudness within an experimental block)^27^. We used here an even larger sound corpus than in previous studies, with 4 different sources for the voice category, 2 male and 2 female, singing 4 different vowels and four different musical instruments (Piano, Saxophone, Bassoon, Clarinet). The large sound corpus was intended to prevent participants from identifying an artificially acoustic cue that would reliably indicate of a given target category, which would not be representative of natural sound recognition outside of the laboratory.

Participants were first tested in a gating experiment, measuring their ability to recognize short vowel and instrument sounds presented in isolation and without time limit to respond. This participant selection experiment was intended to exclude participants who would not produce any meaningful result on the main experiments, where sequences of such short sounds were presented. Four different RASP experiments were run to assess the fastest presentation rate for which target recognition was still possible. In each of these experiments, a symmetric design was used: voice targets to be reported in a stream of musical instruments as distractors and instrument targets to be reported in a stream of voice distractors. In a first set of three experiments, we tested for the effect of single sound duration, number of sounds in a sequence, relative pitch between target and distractors, target position in the sequence on performance. In a fourth experiment, reaction times (RTs) were also measured. Overall, fast presentation rates were found for recognition in all conditions, with an advantage for vocal targets.

## Methods

### Participants

Thirty-eight participants were recruited for this study. Eight of them did not take part in the experiments because they had more than 20 dB HL hearing loss at one or more of the audiometric frequencies between 0.125 and 4 kHz (audiograms performed with an Echodia Elios audiometer). The 30 remaining participants were included in the participant selection experiment described below. Based on the exclusion criteria defined in the Procedure, only 24 of them (14 women; mean age = 24.0 ± 3.2) took part in the main experiments. Eight of the selected participants took part in the ‘very short sounds’ experiment, 7 in the ‘number of sounds’ experiment and 9 in the ‘pitch’ experiment. Then, 14 of the 24 selected participants were again recruited for the ‘RT’ experiment in the 7 months following the first three experiments (8 women; mean age = 24.3 ± 3.1). One of them did not follow the instructions and was excluded, so only 13 participants took part in the RT experiment.

The Institutional Review Board of the French Institute of Medical Research and Health ethically approved this specific study prior to the experiment (Opinion No. 15-211) and all participants provided written informed consent to participate. They were compensated for their participation. All experiments were performed in accordance with relevant guidelines and regulations, as described in the approved protocol.

### Stimuli

Briefly, all stimuli were sequences of very short snippets of natural sounds. Sound samples were extracted from the RWC Music Database^28^. Two sound categories were used: singing voices and musical instruments, with four different sound sources in each category. The voice sounds were two men singing the vowels /a/ and /i/ and two women singing the vowels /e/ and /o/. The instrument sounds were: a bassoon, a clarinet, a piano and a saxophone. All the different sound sources were chosen, as targets, in equal proportion in all the experiments with a pseudo-random selection. Twelve pitches were selected for each sound source, the same for all sources ranging from A3 to G#4.

For the participant selection experiment, the short excerpts of sounds were presented in isolation (gating experiment). Seven sound durations were tested: 2, 4, 8, 16, 32, 64 and 128 ms. The sounds were gated at these durations with a Hanning window. The starting point of the gating window was randomly chosen between 0 and 100 ms from the onset of the sound on each trial. Finally, stimulus intensities were normalized by their root-mean-square level and divided by the square root of their duration^5^.

For the four RASP experiments, sequences of these short-gated sounds were created. How the sequences were constructed is described in the Procedure (see below). For each sequence, gated sounds were generated following the procedure described above (random beginning of the Hanning window, normalization…) and with the same sound corpus.

### Apparatus

Participants were tested individually in a double-walled Industrial Acoustics (IAC) sound booth. Stimuli were presented through a RME Fireface digital-to-analog converter at 16-bits resolution and a 44100 Hz sample-rate. For the ‘RT’ experiment, stimuli were presented through a TDT RM1 Mobile Processor (Tucker-Davis Technologies) digital-to-analog converter at 16-bits resolution and a 48828 Hz sample-rate. They were presented diotically through a Sennheiser HD 650 headphone at a comfortable loudness level (~70 dB A). No time limit was imposed (except for the ‘RT’ experiment, see Procedure) and a visual feedback (green for correct responses, red for incorrect) was provided once the participant had responded.

### Procedure

First, the participant selection experiment was run. All potential participants were tested on their ability to recognize very short sounds presented in isolation. On each trial, participants heard a short sound, which could be either a voice or an instrument. They had to indicate whether the sound was a voice or an instrument (one-interval two-alternative forced-choice task). The two sound categories and the seven sound durations were presented in a randomized order, with equal probability. For each trial, the pitch and the beginning of the gating window were chosen randomly. This contributed to generate a large acoustical variability in the experimental sound corpus, since each short snippet of sound was different on each trial and for each participant. A strict criterion for further inclusion was used: the mean performance minus one standard deviation. This was to ensure that participants could, during the main experiments, recognize a target sound within the RASP sequence: otherwise, they would produce irrelevant results and simply add to the experimental noise. No attempt was made to train the participants that failed the inclusion criterion, even though it is likely that this could have been effective. To reduce fatigue associated to a relatively long and demanding experiment, the number of repetitions used in this participant selection was reduced mid-experiment. The first ten participants performed 44 repetitions for each category and for each sound duration, whereas the next twenty participants performed 24 repetitions. Before the test, the first ten participants were familiarized with the task with a short training period consisting in 28 trials, for which the seven sound durations were presented in a decreasing order and with 4 trials per duration. The twenty following participants performed the same short training phase plus, first, a passive listening of the 8 original sounds with a 250-ms duration. There was no impact of this minor methodological change on the results: t-tests comparing performance before and after the change failed to reject the null hypothesis, for all sound durations (all p > 0.05), indicating that the performances were not significantly different between sub-groups of participants. In addition, two one-sided test (TOST) procedures also indicated that performances were equivalent (p < 0.05) at ± 0.4, in terms of d-prime scores, for the 2, 4 and 128-ms sound durations; at ±0.5 for the 8, 32 and 64-ms sound durations; and at ±0.6 for the 16-ms sound duration. Note finally that this experiment simply served as an inclusion criterion.

For the main RASP experiments (termed below ‘very short sounds’, ‘number of sounds’, ‘pitch’ and ‘RT’), sequences of short natural sounds were presented in rapid succession. Each short sound was gated with the same procedure as the one described in the participant selection experiment; the same sound database was used, with singing voices and instruments sounds. For all experiments, the task was similar: participants heard a sequence of short sounds and had to decide whether a sound from the target category was present in the sequence, or not (yes/no task). In 50% of the trials (the ‘no’ trials), sequences were composed of distractor sounds only; in the other 50% (the ‘yes’ trials), one target sound was embedded in the sequence, in a random position with the only constraint that the target sound could not be in the first and last positions of the sequence. The proportion of the yes and no trials was different for the RT experiment (see below). Target and distractors were alternatively voice or instrument sounds, for each experiment, in counterbalanced order. The presentation rate of the sequence (from slow to very fast sequences) was also varied through all three experiments and was the main experimental measure.

The ‘very short sounds’ RASP experiment was performed to investigate fastest presentation rates for sound recognition, while limiting the potential acoustic cues to timbre ones: pitch was randomly varied from sound to sound in the sequences and all individual sound durations were the same: 16 ms. The ‘number of sounds’ and ‘pitch’ experiments were carried out to test for two potential interpretations of the results.

Firstly, the drop in performance with an increased presentation rate could be due to memory limitations rather than a reduced available time to analyze each sound in the sequence. This is because, with fixed-duration sequences, the number of sounds increased as the rate increased. In the ‘number of sounds’ experiment, fixed-durations sequences (500-ms) were used together with fixed-number of sounds sequences (7 sounds). Note that with a fixed sequence duration and the given presentation rates (cf. Table 1), for the 16-ms sound duration, the number of sounds in the sequences varied between 3 (at 5.3 Hz) and 30 (at 60 Hz); for the 32-ms sound duration, it varied between 3 (at 5.3 Hz) and 15 (at 30 Hz). The sequences with a fixed number of sounds (only with the 32-ms sound duration) varied between 1.321 s (at 5.3 Hz) and 0.233 s (at 30 Hz). Moreover, the increase in sound duration (16 to 32 ms) aimed at avoiding a floor effect in the recognition performances, which we expected because of the difficulty of the task with the very high presentation rates of the fixed sequence duration task.

**Table 1:** Tested conditions in the main experiment with sequences of short sounds presented rapidly.

Conditions	RASP experiments			
	‘very short sounds’	‘number of sounds’	‘pitch’	‘RT’
Sound duration	16 ms	32 ms	32 ms	32 ms
Sequence type	Fixed duration	Fixed duration vs. fixed number of sounds	Fixed number of sounds	Fixed number of sounds
Pitch	Randomized	Randomized	Randomized vs. fixed	Randomized
Presentation rate	5.3, 7.5, 10.6, 15, 21.2, 30 and 60 Hz	5.3, 7.5, 10.6, 15, 21.2 and 30 Hz	5.3, 7.5, 10.6, 15, 21.2 and 30 Hz	5.3, 7.5, 10.6, 15, 21.2 and 30 Hz

Secondly, performance could have been limited by forward masking, as even partial masking would distort the spectrum of individual sound in the sequence. To test for this, we compared, in the ‘pitch’ experiment, random-pitch sequences, where each individual sound in the sequence had a randomly selected pitch, with fixed-pitch sequences, where the pitches of every sound in the sequence were the same (while varying from sequence to sequence). An interpretation based on forward-masking would predict worse results for the fixed-pitch sequences, because of a much larger frequency overlap between successive sounds. A summary of the different conditions used for each experiment is given in Table 1.

For the ‘very short sounds’ experiment, participants performed 44 repetitions for each target category (Voices, Instruments) and for each presentation rate. All participants performed both types of blocks (voices as a target and instruments as a target), but in a counterbalanced order between them. Presentation rates (from 5.3 Hz to 60 Hz; see details in Table 1) were randomized within each block. Before each category block, the participants were accustomed with RASP sequences by listening to sequences with longer individual sound durations (64 ms then 32 ms) and a presentation rate increasing progressively from 5.3 to 15 Hz (64 trials in total). Then, they performed 112 trials identical to the main experiment test to get acquainted with the recognition task. The results of this familiarization phase were discarded.

For the ‘number of sounds’ and the ‘pitch’ experiments, participants performed 24 repetitions for each category, for each presentation rate and for each condition: fixed duration/fixed number of sounds and randomized/fixed pitch respectively. As for the first ‘very short sounds’ experiment, presentation rates (from 5.3 Hz to 30 Hz; see details in Table 1) were randomized within each block. For the ‘number of sounds’ experiment, four types of blocks were possible: target Voice with ‘fixed duration’ sequences, target Voice with ‘fixed number of sounds’ sequences, target Instrument with ‘fixed duration’ and target Instrument with ‘fixed number of sounds’. These four conditions were counterbalanced between participants. A similar counterbalanced presentation of blocks was performed for the ‘pitch’ experiment (including the ‘random-pitch’ and the ‘fixed-pitch’ conditions). A similar training phase as for the first ‘very short sounds’ experiment was conducted before the ‘number of sounds’ and ‘pitch’ experiments.

For the RT experiment, participants first performed a gating RT experiment, with individual sounds only. Two blocks of a go/no-go task were run in a counterbalanced order: the target was either a voice or an instrument whereas distractors were chosen in the opposite category. On each trial, participants heard a short 32-ms sound and had to press a response-button as rapidly and accurately as possible if they recognized the target; they had to withhold their response in the case of a distractor. Targets were presented in 80% of the trials (the ‘go’ trials) and distractors in 20% of the trials (the ‘no-go’ trials), as is common in RT tasks. Participants were instructed to keep the index finger of their dominant hand on the response button during all the experiment. They performed 48 repetitions for each category. Before the test, participants were familiarized with the task with a short training consisting of 40 trials: the first half with a 250-ms sound duration, the second half with a 32-ms sound duration.

After this first gating RT, participants then performed the RASP RT experiment. Similarly as for the gating RT, participants performed two blocks of a go/no-go task in a counterbalanced order: the target was either a voice or an instrument whereas distractors were chosen in the opposite category. They had to respond as quickly and accurately as possible when they heard a target sound embedded in a sequence of distractors. Targets were present in the sequence 80% of the time. For each target category, they performed 3 separate sub-blocks of 120 randomized trials. They performed in total 48 repetitions for each category and each presentation rate. Before the test, participants were familiarized with the task with a short training consisting of 40 trials: the first half with a 5.3-Hz presentation rate, the second half with a 15-Hz presentation rate.

The ‘very short sounds’, ‘number of sounds’ and ‘pitch’ experiments lasted about 2 hours and half in total. The ‘RT’ experiment lasted about 1 hour.

### Statistical analyses

For all experiments, d-prime scores were computed as a measure of performance^29^. Further analyses were all performed on these d-prime scores. Normality was tested using the Shapiro-Wilk test. We conducted several repeated-measures ANOVA. Whenever the sphericity assumption was violated, the degrees of freedom were adjusted and reported using the Greenhouse-Geisser epsilon correction. When necessary, Tukey-HSD post-hoc tests were conducted. When t-tests are reported, normality conditions were checked. The main statistical analyses are detailed in the text.

## Results

### Participant selection experiment

**Figure 1:**
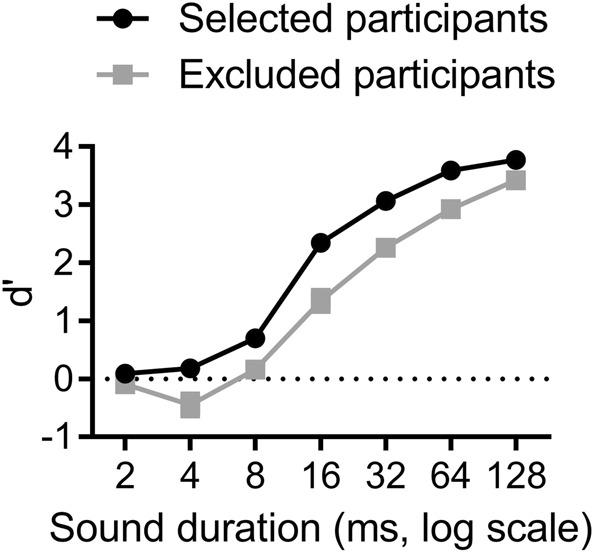
Recognition of individual short sounds (participant selection experiment). Error bars represent the standard errors of the means (too small to be visible in the graph). Performance, as measured by d-prime, increased as the sound duration increased for both the selected group (black curve) and the excluded group of participants (grey curve).

In order to select the participants on their ability to recognize a very short sound presented in isolation, we pooled the d-primes on the seven durations tested (from 2 to 128 ms) and computed the mean and standard deviation: 1.84 and 0.32 d-prime units, respectively. Six participants were below the criteria fixed for selection, i.e. mean minus one standard deviation: their mean d-prime was of 1.37. Results are represented in Fig. 1, with the d-prime scores as a function of duration for each group of participants (the 24 selected and the 6 excluded).

Results for the selected group of participants are very similar to those already reported^5^: performance increased as the sound duration increased [F(6, 138) = 348.3; p < 0.00001; η_p_^2^ = 0.94] and sound could be recognized significantly above chance for durations as short as 4 ms [t(23) = 2.19; p < 0.04].

All following RASP experiments were conducted with these 24 participants, who showed a sufficient ability to recognize short sounds presented in isolation so as to be meaningfully tested with sequences of such short sounds.

### ‘Very short sounds’ experiment

**Figure 2:**
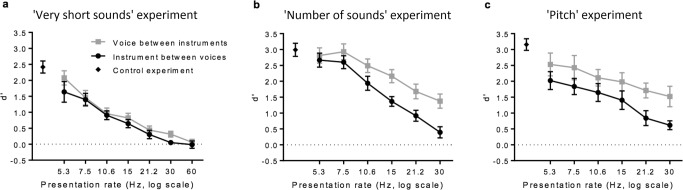
RASP performances: recognition of a short target in a sequence of short distractors presented rapidly. Mean d-prime scores are plotted for each experiment condition as a function of presentation rates. The error bars represent the standard errors of the means. Results from the control experiment, when short sounds were presented in isolation, are represented by a diamond on the left of the curves. For all panels, performance linearly decreased (on a log scale) as the presentation rate increased and voice were better recognized within instruments than the reverse. Panel a: Performance for sequences composed of 16-ms sounds. Panels b and c: Sequences of 32-ms sounds were presented. Each line is an average of the ‘fixed duration’ and the ‘fixed number of sounds’ conditions and of the ‘random pitch’ and the ‘control pitch’ conditions respectively. There was respectively no difference between these conditions for all presentation rates.

Results are represented in Fig. 2a. A repeated-measures ANOVA was performed on the d-prime scores with presentation rate and sound target as within-subjects factors. As expected, recognition performance decreased as presentation rate increased [F(6, 42) = 29.590, p < 0.00001, η_p_^2^ = 0.809]. Least-squares linear regressions were used to investigate whether the decreases in performance with presentation rate were linear. Separate regression lines were performed on the average performances over participants, for each sound target condition. For both sound target conditions, the decrease was linear on a log-scale [instrument target: R² = 0.97; voice target: R² = 0.94; the slopes were significantly non-zeros: p < 0.001]. In addition, the ANOVA revealed better performances for a voice target embedded in a sequence of instruments than the reverse [F(1, 7) = 7.901, p < 0.03, η_p_^2^ = 0.530]. However, the gain in d-prime for the voice target was, on average, relatively small: Δd-prime = 0.2.

To determine the maximum rate for recognition of a target within the sequence, performance for a given rate were compared to chance level (d-prime = 0). An instrument target could be recognized within a sequence of voice distractors up to sequences of 21.2 Hz [at 15 Hz: t(7) > 5, p < 0.002; at 21.2 Hz: t(7) = 2.289, p = 0.056; at 30 Hz and 60 Hz, respectively: t(7) = 0.645, p = 0.539 and t(7) = −0.100, p = 0.924]. For a voice target within a sequence of instruments distractors, recognition was possible up to 30 Hz [t(7) > 3, p < 0.02; at 60 Hz: t(7) = 0.651, p = 0.536]. These results indicate successful recognition with very fast rates of presentation, with a recognition of voice targets at even higher rates than instrument targets. This again suggests better recognition for the voice category than the instrument one.

Finally, to investigate a potential difference between the slowest presentation rate and the sound presented in isolation, we compared the d-primes obtained in the participant selection experiment with the d-primes obtained for the 5.3 Hz rate. There was a marginally significant difference [t(7) = 2.290, p = 0.056]. A TOST procedure indicated that performances are equivalent (p < 0.05) at ±1.2 in terms of d-prime scores, showing that at least for sufficiently slow presentation rates, no information was lost, compared to the isolated sound presentation.

### ‘Number of sounds’ experiment

Results are represented on Fig. 2b. The repeated-measures ANOVA with presentation rate, sound target and the ‘number of sounds’ conditions as within-subjects factors revealed, as in the first experiment, a decrease in performance with an increase in presentation rate [F(2.06, 12.36) = 71.43; p < 0.0001, η_p_^2^ = 0.923]. There was no main effect of the sequence type [F(1, 6) = 0.48, p = 0.51, η_p_^2^ = 0.07] and no interaction effect between the sequence type and the presentation rate [F(2.62, 15.73) = 3.09, p 0.06, η_p_^2^ = 0.34]. The ANOVA also revealed on average a better recognition for a voice target between instrument distractors than the reverse [F(1, 6) = 10.02, p < 0.02, η_p_^2^ = 0. 63]. The two-way interaction between sequence type and sound target category was not significant, nor the three-way interaction [resp.: F(1, 6) = 2.65, p = 0.15, η_p_^2^ = 0.31; F(3.65, 21.92) = 2.07, p = 0.1, η_p_^2^ = 0.26].

The two-way interaction between sound target category and presentation rate was significant [F(3.15, 18.91) = 3.52, p < 0.05, η_p_^2^ = 0.37]. This interaction was due to similar performances between voice and instrument targets for the slowest presentation rates, whereas voice target outperformed the instrument target for the faster presentation rates [p = 0.99 at 5.3 Hz; p = 0.7 at 7.5 Hz; p = 0.08 at 10.6 Hz, p < 0.01 for 15 and 21.2 Hz; p < 0.001 at 30 Hz].

Due to this significant interaction, the linear regressions were conducted on the average performances only from the 7.5 Hz to the 30 Hz presentation rates. As in the first experiment, for both categories, performance linearly decreased on a log scale as a function of presentation rate [instrument target: R² = 0.99; voice target: R² = 0.99; the slopes were significantly non-zeros: p < 0.001].

In this experiment, the target was detected above chance for all presentation rates [t(6) > 4, p < 0.005], except in the case of an instrument target in sequences with a fixed duration, at 30 Hz [t(6) = 1.099, p = 0.314].

Finally, as in the ‘very short sounds’ experiment, there was no significant difference in performances between the 5.3-Hz condition and the sound presented in isolation [t(6) = 1.44, p = 0.2]. A TOST procedure indicated that performances are equivalent (p < 0.05) at ± 0.7 in terms of d-prime scores.

### ‘Pitch’ experiment

Results for the pitch experiment are plotted on Fig. 2c. The repeated-measures ANOVA conducted with presentation rates, sound target category and pitch conditions as within-subjects factors again found a decrease in performances with an increased presentation rate [F(5, 40) = 26.38, p < 0.00001, η_p_^2^ = 0.77] and a better recognition for a voice target than an instrument target [F(1, 8) = 9.12, p < 0.05, η_p_^2^ = 0.53]. Moreover, there was no effect of the pitch condition: the main effect of the pitch condition was not significant, nor the two-way interactions with sound target category and with presentation rate [resp.: F(1, 8) = 0.55, p = 0.48, η_p_^2^ = 0.07; F(1, 8) = 0.002, p = 0.98, η_p_^2^ = 0.0; F(5, 40) = 1.99, p = 0.1, η_p_^2^ = 0.2]. The two-way interaction between sound category and presentation rate and the three-way interaction were not significant either [resp.: F(5, 40) = 1.33, p = 0.27, η_p_^2^ = 0.14; F(5, 40) = 0.2, p = 0.96, η_p_^2^ = 0.02]. The mean performance decreased linearly on a log scale as presentation increased [instrument target: R² = 0.96; voice target: R² = 0.99; the slopes were significantly non-zeros: p < 0.001]. The target was recognized above chance for all presentation rates in all conditions [t(8) > 3, p < 0.02]. Finally, performances in the 5.3 Hz condition and in the individual sound condition were significantly different [t(8) = 3.66, p < 0.007], probably linked to a general downwards offset on the RASP performance for this experiment, maybe due to the participants’ fatigue.

### Effect of the target position in the sequence

To investigate potential memory effects, with a possible better performance for a target position close to the beginning or the end of the sequence^30^, we analyzed an a-posteriori effect of the target position in the sequence. To increase statistical power, we performed a pooled analysis on the blocks in which sequences had a fixed number of sounds (‘number of sounds’ and ‘pitch’ experiments).

A repeated-measures ANOVA was performed on the d-prime scores with the sound target categories and the five target positions as within-subject factors and the experiment as a categorical factor. Performances were equivalent in both experiments [F(1, 14) = 0.43, p = 0.52, η_p_^2^ = 0.03]. As assessed previously, voice targets were better recognized than instrument targets [F(1, 14) = 12.14, p < 0.004, η_p_^2^ = 0.46]. More importantly here, the effect of the target position was not significant as a main effect (on average, d-primes for each target position varied between 1.42 and 1.77), nor in the two-way interactions with experiment and with sound category [resp.: F(4, 56) = 0.71, p = 0.59, η_p_^2^ = 0.048; F(4, 56) = 1.09, p = 0.37, η_p_^2^ = 0.072; F(4, 56) = 1.13, p = 0.35, η_p_^2^ = 0.075]. The two-way interactions between experiment and sound category and the three-way interaction were not significant either [resp.: F(1, 14) = 0.079, p = 0.78, η_p_^2^ = 0.006; F(4, 56) = 1.89, p = 0.12, η_p_^2^ = 0.12].

### ‘RT’ experiments

#### Data transformation and analyses

Only the RT corresponding to the hits were taken into account in the analyses. The RT below 100 ms were considered as errors (anticipation; 1.2% of the hits in the RASP experiment). The RT distributions were log-transformed for each participant and for each condition (the log-normality was verified with Kolmogorov-Smirnov tests): each distribution was transformed to normal distributions (with logarithm function); the mean of each normal distribution was computed; and converted back to milliseconds with exponential functions for more easily interpretable reports. Statistics were then performed on these transformed data.

#### Gating RT experiment

**Figure 3:**
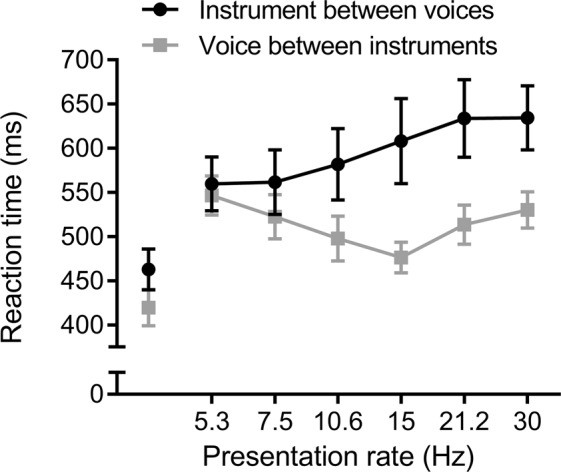
Recognition of a target presented in isolation or in a RASP sequence evaluated with RTs: faster RT for voices. Mean RTs (in ms) for both instrument and voice targets. The results for isolated targets in the gating experiment are represented as single points on the left of the curves. The error bars represent the standard errors of the means.

Participants recognized well and similarly (mean d-prime = 2.74) short voice and instrument sounds when presented in isolation, in a standard gating paradigm [paired-samples t-test: t(12) = −1.61, p = 0.13; Δd-prime = 0.38]. However, in terms of RTs, responses were significantly faster for voice targets than for instrument targets [paired-samples t-test: t(12) = 3.59, p < 0.01; ΔRT = 43 ms; see Fig. 3].

#### RASP RT experiment

Figure 3 represents the results in terms of RTs corresponding to the hits, as a function of the presentation rate.

We first tested whether the results obtained were obtained here to the results as in the first RASP experiments in terms of accuracy, as measured by d-primes. A repeated-measures ANOVA was performed, with the d-prime score as the dependent variable and the sound category and presentation rate as within-subjects factors; it confirmed very similar effects as obtained previously. As in the previous RASP experiments, performances decreased significantly with an increased presentation rate [F(5, 60) = 21.666, p < 0.00001, η_p_^2^ = 0.644]. Furthermore, we observed a better recognition of a voice target between instrument distractors than the reverse [F(1, 12) = 14.314, p < 0.003, η_p_^2^ = 0.544; Δd-prime = 0.71]. The two-way interaction was not significant [F(5, 60) = 1.036, p = 0.405, η_p_^2^ = 0.080].

More importantly here, we also performed a repeated-measures ANOVA with the transformed RT as the dependent variable and the sound target category and presentation rate as within-subjects factors. Responses for a voice target in a sequence of instruments were on average faster than for an instrument target [F(1, 12) = 8.67, p < 0.05, η_p_^2^ = 0.420; ΔRT = 82 ms]. There was no main effect of the presentation rate [F(2.22, 26.68) = 1.68, p = 0.2, η_p_^2^ = 0.12] and no significant effect of the two-way interaction [F(2.13, 25.60) = 2.69, p = 0.08, η_p_^2^ = 0.18].

The non-conformity of the sphericity assumption was probably due here to large differences in terms of variance between instrument and voice category (see Fig. 3). We thus analyzed this variance in more details, with a repeated-measures ANOVA with the target sound category as a within-subjects factor and the inter-participants variance at each presentation rate as the dependent variable. It clearly revealed that the variance of the results was much higher for instrument targets than for voice, pointing, again, towards a processing difference and a specificity of the voice.

Finally, there was a significant difference between the RT for the recognition of an individual sound compared to its recognition in a RASP sequence at the slowest (5.3 Hz) presentation rate [instruments: t(12) = −2.567, p < 0.03; voices: t(12) = −3.691, p < 0.004], showing that, in general, the RASP task was more demanding in terms of cognitive resources than the gating task. This was also reported informally by participants during the post-experiment debriefing.

## Discussion

This series of experiments provides two novel findings related to the time course of auditory recognition of natural sounds. Firstly, the results showed that auditory recognition is extremely fast, as evidenced by the highest presentation rate up to which participants could still recognize a target sound embedded in a sequence (30 Hz). Crucially, this rapid processing does not depend on the details of the RASP design, as it was found for several variants (fixed number of sounds vs fixed sequence duration, fixed pitch across sounds vs random pitch, target toward the beginning of the sequence vs target toward the end of the sequence). Secondly, a robust voice effect was found: voice targets within an instrument sequence were better recognized and faster, at all rates than instrument targets in a voice sequence.

For all experimental conditions, short sounds could be recognized reasonably well when presented individually (d-prime around 2.5 and 3 for short 16-ms and 32-ms sounds, respectively). This was partly due to our inclusion criterion, but also consistent with previous reports^5^. When presented in rapid sequences, with a single target embedded in a series of multiple distractors, recognition performance for these same short sounds linearly decreased, on a log scale, as the presentation rate increased. Recognition performance was still above chance for the fastest rate tested, 30 Hz, for voice sounds and it was above or just at chance at 30 Hz for instrument sounds. As shown extensively in vision research with the RSVP paradigm^11,31^, perceiving and attending to the stimulus N + 1 interferes with the processing of the stimulus N. Thus, the shortest time interval between stimulus N and N + 1 is a measure for the time window of processing of the stimulus N, in any given task. From our data, we can specify the time window for sound recognition based on timbre, in the auditory modality. Our data provide an upper limit, which yields the best recognition performance with no loss of information compared to sounds presented in isolation and a lower limit, for which the recognition was above chance, which defines the shortest time for a first ‘read-out’ of the information by the auditory system.

The upper limit was found to be around 200 ms, corresponding to the slowest presentation rate tested of 5.3 Hz. This is the time window required for equal performance for sounds presented in sequences and for sounds presented in isolation. This upper limit is broadly consistent with the time course of recognition suggested with other paradigms^20^. Note however that the results reported in Massaro’s studies^20^ were derived from stimuli with considerably less acoustical variability (tones and very few examples of speech syllables) than the corpus used in the present study (many sound sources, randomly gated for each trial and participant, varied in pitch). Massaro predicted longer processing time for natural stimuli. Interestingly, this is not what we observed. Results were also broadly consistent with the estimation obtained based on recognition RTs on a similar (but again less diverse) set of stimuli^2^.

The lower limit, estimating the minimal processing time required to obtain above-chance performance, was found to be 33 ms, corresponding to the 30-Hz presentation rate. This short time window is also in accordance with some estimates of temporal processing in hearing, but with very different paradigms. Massaro^20^ proposed the concept of a preperceptual store, from which a read-out could be performed, in order to recognize a stimulus. He showed that the read-out started as the stimulus was still ongoing, as early as 30 ms, with similar levels of performance achieved (d-prime around 1). Using a procedure sharing some features with RASP (rapid sequences of tones from which a target had to be reported, defined by a combination of frequency, location and intensity values), it has been proposed that different perceptual features of sounds could be processed in parallel^32^. Here, one interpretation of the lower limit result could also be a parallel processing of complex features extracted from the natural sounds^33^. Recognition is not fully completed for all features by 30 ms, but enough time has passed for some identifying features of voices and intruments to be computed. Although this may be unrelated, it should also be mentioned that similar time constants of about 30 ms have been found for pitch perception^17^ and synchrony detection across perceptual streams^34^.

Crucially, here we could test several alternative interpretations for the RASP results and rule out factors unrelated to speed of processing. Firstly, the decrease in performance as the presentation rate increases in the main RASP experiment could have been due to an increase of information to be processed by the participant, unrelated to speed of processing: with a fixed-duration sequence, as we originally used^14^, the number of sounds increases with rate. Here, the ‘number of sounds’ experiment showed that this is not a valid interpretation, as fixed number of sound and fixed duration sequences produced similar performance. Secondly, forward masking could have affected performance in the faster sequences, as even partial masking of harmonics of one sound by harmonics of a previous sound could have changed the spectral shape of sounds when in sequence. The ‘pitch’ experiment showed that this is unlikely to be the cause of the performance drop as presentation rate increases, because we did not observe better performance for random-pitch conditions, which minimized forward masking across sounds in the sequence and constant-pitch conditions, which maximized forward masking. Finally, the absence of effect of the target position in the sequence on the performance shows that the RASP effect was not even partly due to classic sequential memory effect, with better recall of first and last items in a sequence^35^.

Another finding emerged from the data: voice targets were processed more efficiently than instrument targets. This voice advantage was found both in terms of accuracy, with a better recognition performance for voices as measured by d-prime and in terms of speed of processing, with faster RTs for voices and better performance at fast presentation rates. Given our stimulus set, which was diverse and carefully balanced and given the symmetric nature of the design, we believe that there is no trivial acoustical explanation for the voice advantage. The acoustical variability of the sound set was large for both categories and listeners were left only with timbre cues to perform the task (similar loudness cues and random pitches within an octave range, with no possibility to use the pitch as a cue). The voice sounds used as a target in the voice blocks were the same sounds used as voice distractors in the instruments blocks (and vice versa). Moreover, modeling on a similar set of sound failed to show any relation between acoustic similarity and RTs^2^.

The voice specificity, however, seems consistent with a large number of neuroimaging studies, which provided evidence for a specific voice treatment in auditory cortex^26,36–42^. It should be noted that selective activations of cortical regions have also been identified for other auditory categories, like man-made objects or instruments^25,43,44^, but the case of the voice is particularly well documented and seems to make a consensus. The predominant selectivity to the voice has been explained by the social necessity to extract information on an individual (e.g. gender, identity, emotional state)^45^, or by virtue of being the most familiar sounds for human listeners^46^. There are only few behavioral studies suggesting distinctive property of vocal sounds, but so far they seem to show a similar trend as in the current study. Comparing voice and instrument stimuli, faster RTs were observed for voice stimuli^2^. With the same corpus of sounds, but this time presenting to the participants only short snippets of sounds (like in the present control experiment), it was shown that the minimal duration necessary to recognize a voice sound was lower (4 ms) than for an instrument sound (8 ms)^5^.

The voice advantage could also be thought of as a pop-out effect. A similar asymmetry was obtained^47^ in the recognition of basic features in auditory sequences with, in particular, a better recognition of frequency-modulated targets between pure tone distractors than the reverse. The interpretation for this finding was that frequency modulation could be coded as an extra feature which consequently drives the recognition of the target. Here, it could be argued that the voice popped out of the instrument distractors, because there is neural selectivity to specific voice features. The fact that the voice effect did not depend on the number of distractors (comparison, for a given presentation rate, of the ‘fixed number of sounds’ condition with the ‘fixed sequence duration’ condition) would be an additional argument in favor of a pop-out effect. The voice features are probably located in conjoint temporal and spectral modulations, which can characterize natural sounds like vocalizations or environmental sounds^48^.

Strikingly, the shortest processing times we observed (30 ms for voices) were almost as short as some estimates of the time it takes for neural information to reach auditory cortex, which varies around 15–30 ms^49–51^. An EEG study has also suggested that differential processing of categories of complex sounds could be observed in event-related potential with a latency of 70 ms^25^. An important point to keep in mind is that our operational definition of recognition could be subserved by different processes: a match of the target sound with a previously-known template of the target category; a match of the target sound with a template acquired during the experiment; or a holistic judgment on the sequences. While we cannot decisively arbiter between the first two interpretations, we can confidently rule out the last one because of the trial-to-trial variability of our sequences. By using unique snippets of sounds to build unique sequences for each trial and listener and by varying these sounds in terms of pitch and sound source, it is hard to imagine how reliable sequence-wide cues could have been available. Rather, we likely taped into processes that are useful for recognizing natural sounds in ecological situations. Thus, the fact that the behavioral data demonstrated processing times at the lowest bound or even lower than predicted by neural data is remarkable and it suggests an impressive efficacy in the use of neural cues for timbre recognition.

To summarize, the RASP paradigm seems to be a robust and useful way to estimate processing times in audition. When used here to probe the processing time for sound recognition based on timbre, using several variants of the paradigm, we always observed an impressively rapid processing speed, right down to the lowest limit of what would be possible as predicted by neural data and with an advantage for ecologically important sounds such as the voice.
